# Pediatric Stroke Associated With a Rare Pathogenic PKP2 Variant: A Diagnostic Challenge

**DOI:** 10.7759/cureus.102553

**Published:** 2026-01-29

**Authors:** Alaa Salman, Mohamed O E Babiker, Maria Khan, Nidheesh Chencheri

**Affiliations:** 1 Pediatric Neurology, Al Jalila Children's Specialty Hospital, Dubai, ARE; 2 Neurology, Mohammed Bin Rashid University of Medicine and Health Sciences, Dubai, ARE; 3 Neurology, Rashid Hospital, Dubai, ARE

**Keywords:** arrhythmogenic right ventricular dysplasia, diagnostic and therapeutic challenge, genetic diagnosis, pediatric stroke, pkp2 variant, plakophilin-2 (pkp2) mutation

## Abstract

Pediatric ischemic stroke is an uncommon but serious neurological condition with highly variable etiologies. Cerebellar infarctions are particularly rare in children and often present with nonspecific symptoms, contributing to delays in diagnosis. We report a case of a previously healthy 15-year-old female who presented with acute worsening headache, dizziness, vomiting, dysarthria, and left-sided neurological deficits. Neuroimaging revealed an acute non-hemorrhagic infarct in the left cerebellar hemisphere within the superior cerebellar artery territory. Given her low National Institutes of Health Stroke Scale (NIHSS) score, established infarction on MRI, and absence of a diffusion-FLAIR (fluid-attenuated inversion recovery) mismatch, mechanical thrombectomy was deferred. Standard evaluation, including echocardiography, Holter monitoring, hemoglobin electrophoresis, and thrombophilia testing, did not identify an underlying etiology. Anticoagulation was initiated due to concern for a potential cardioembolic mechanism in the setting of posterior circulation involvement, despite the absence of a documented arrhythmia. Whole-exome sequencing subsequently revealed a heterozygous pathogenic PKP2 variant associated with arrhythmogenic right ventricular dysplasia (ARVD). Cardiology evaluation, including ECG, echocardiography, and cardiac MRI, demonstrated no structural or functional abnormalities, and the patient did not meet diagnostic criteria for ARVD. She was managed with antithrombotic therapy and demonstrated gradual clinical improvement.

This case illustrates the diagnostic complexity of pediatric stroke when routine investigations fail to identify a cause and highlights the role of genetic testing in informing surveillance rather than establishing causality. Although the cerebrovascular relevance of PKP2 variants remains uncertain, their association with arrhythmogenic cardiac disease prompted ongoing cardiac follow-up and family screening. Further research is needed to clarify whether desmosomal gene variants have any independent relevance to cerebrovascular risk.

## Introduction

Arrhythmogenic right ventricular dysplasia (ARVD) is a rare cardiovascular disease that predisposes to ventricular arrhythmias, potentially leading to sudden cardiac death. The exact prevalence is unknown, but it is thought to affect six in 10,000 people. An incidence of 44 per 10,000 people has been reported in certain Mediterranean and southern US populations. It is the second leading cause of sudden cardiac death in young adults, after hypertrophic cardiac disease [1].

ARVD occurs due to fibrous-adipose tissue replacement of the right ventricular muscle, leading to arrhythmias. Patients usually have abnormal echocardiographic findings; however, early subtle findings of right ventricular dysfunction can be missed on echocardiography, necessitating further imaging such as cardiac magnetic resonance imaging (MRI). There are several mutations associated with ARVD, one of them being a mutation in plakophilin-2 (PKP2) that leads to impaired desmosomal function, resulting in ARVD [2].

Although ARVD typically manifests in adolescence or early adulthood, the detection of a PKP2 mutation in asymptomatic children is becoming more frequent with the expanding use of genetic screening. The relationship between PKP2 variants and thromboembolic or cerebrovascular events remains poorly understood, as major cohort studies [3] do not identify stroke as an established manifestation.

Any potential association is therefore speculative and may be mediated through indirect mechanisms such as occult arrhythmias or early myocardial dysfunction rather than direct vascular pathology.

We present a case of a previously healthy adolescent female who developed an acute posterior circulation stroke and was subsequently found to carry a heterozygous pathogenic PKP2 variant in the absence of any cardiac manifestations. This case highlights the diagnostic challenges in pediatric stroke, raises questions regarding the potential cerebrovascular implications of PKP2 mutations, and underscores the importance of comprehensive evaluation, including genetic testing, when no clear etiology is identified.

## Case presentation

A previously healthy 15-year-old female presented to our hospital with complaints of headache, dizziness, and vomiting. She reported a mild left-sided headache for three to five days prior to presentation. Six hours before arrival, she developed a sudden, severe exacerbation of the headache on the left side, associated with dizziness and three episodes of vomiting. Neither she nor her family reported any limb weakness.

She was born at term following an uncomplicated pregnancy and delivery, with an unremarkable postnatal course. She had no known medical conditions and was not taking any regular medications. She denied a history of chronic headaches. Family history was notable for parental consanguinity and migraine in a maternal aunt. Her siblings were healthy, and there was no family history of sudden cardiac death, syncope, or strokes.

On arrival at the emergency department, she appeared anxious and uncomfortable, with dysarthric speech. Her vital signs were stable, and her Glasgow Coma Scale (GCS) score was 15/15. GCS is a standardized and freely available assessment tool for clinical and research use [3]. Neurological examination revealed a left upper motor neuron facial palsy, left pronator drift, and weakness in the left upper and lower limbs (power 4/5). Strength in the right upper and lower limbs was normal (5/5). She demonstrated left-sided limb ataxia on finger-to-nose and heel-to-shin testing. Reflexes were 1+ on the right and difficult to elicit on the left. She veered and fell to the left during ambulation attempts.

**Figure 1:**
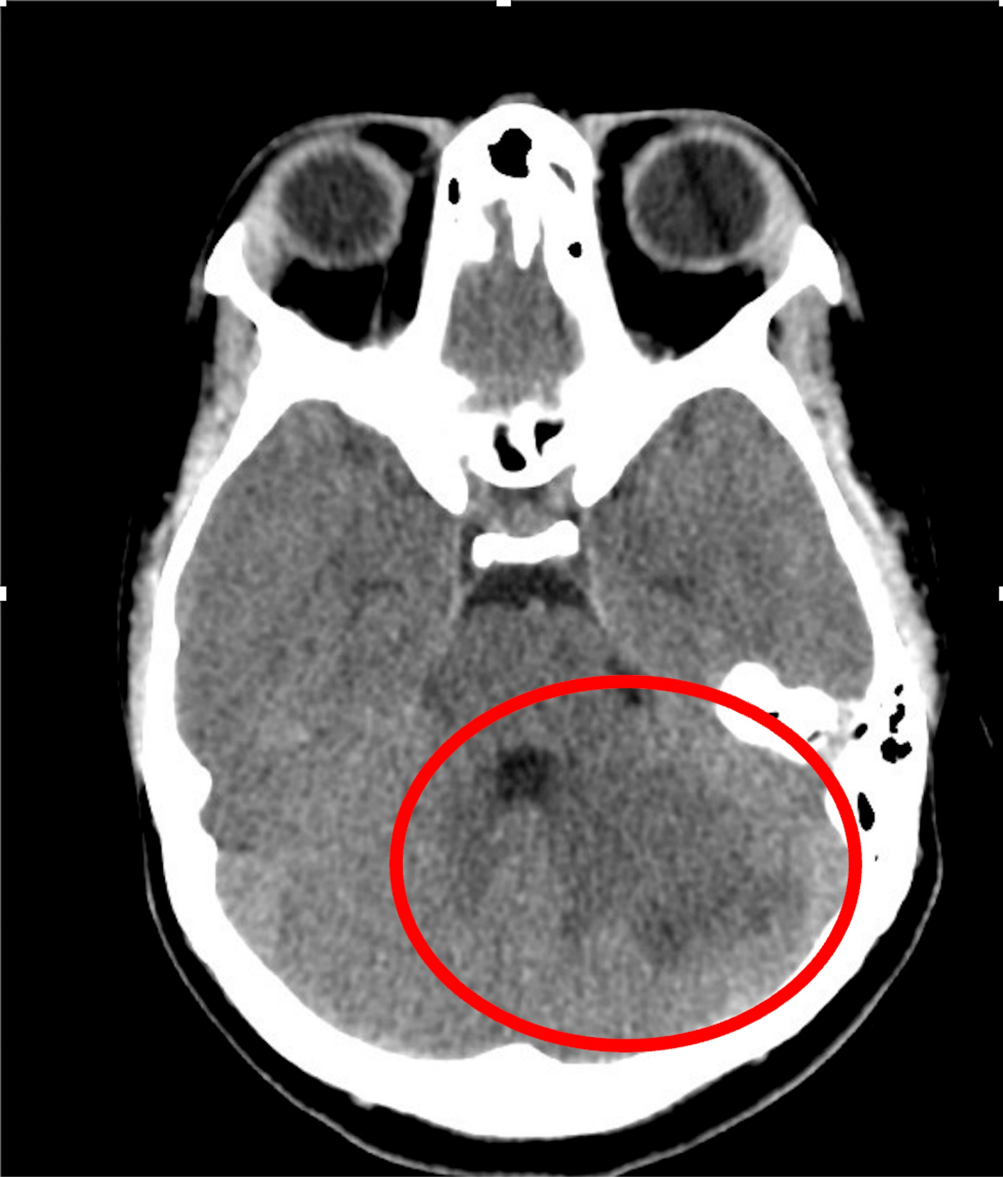
CT of the brain, axial view, showed an area of hypo-density in the left cerebellar hemisphere in the supero-lateral aspect, representing an infarct.

**Figure 2:**
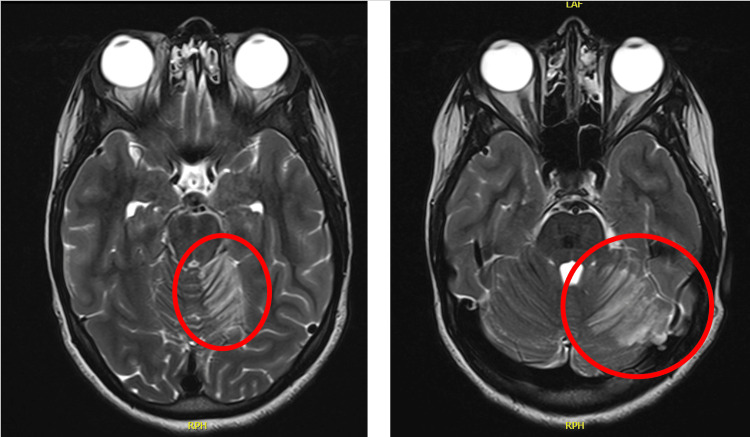
T2-weighted, turbo spin echo MRI of the brain showed T2 hyperintensity in the superolateral aspect of the left cerebellar hemisphere and left medial temporal lobe, suggestive of a non-hemorrhagic infarct in the left superior cerebellar artery territory.

**Figure 3:**
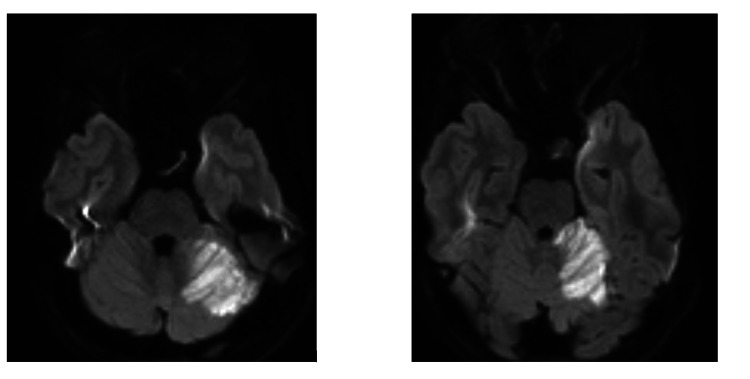
Diffusion-weighted imaging showed diffusion restriction in the territory of the left superior cerebellar artery, confirming an infarct.

An urgent non-contrast CT of the brain (Figure 1) demonstrated hypodensity in the left cerebellar hemisphere, raising concern for infarction versus cerebellitis. Subsequent MRI of the brain (Figures 2, 3) and magnetic resonance angiography (MRA) of the brain revealed an acute non-hemorrhagic infarct in the left cerebellar hemisphere and medial temporal lobe within the superior cerebellar artery territory.

She was transferred to a tertiary stroke center for consideration of mechanical thrombectomy. However, given her mild neurological deficits with a National Institutes of Health Stroke Scale (NIHSS) score of 3 (NIHSS is a publicly available clinical assessment tool that is free for clinical and research use and does not require licensing for clinical or academic use) [4], no DWI/FLAIR (fluid-attenuated inversion recovery) mismatch on MRI brain consistent with established stroke, and a non-occlusive thrombus, thrombectomy was not offered. She was loaded with aspirin 300 mg and admitted for stabilization and neurological monitoring.

She subsequently underwent a routine stroke workup, including complete blood count, renal and liver function tests, electrolytes, coagulation profile, and inflammatory markers, all of which were within normal limits (Table 1).

**Table 1:** Laboratory investigation done at presentation to the hospital showed a normal laboratory stroke workup, excluding common hematologic, inflammatory, metabolic, and prothrombotic causes of pediatric ischemic stroke. Reference ranges are based on standard pediatric laboratory values used at our institution.

Laboratory test	Patient result	Reference range
Hemoglobin (Hb)	11.8 g/dL	11.5–15.5 g/dL
White blood cell count (WBC)	5.8 ×10⁹/L	4.5–13.5 ×10⁹/L
Platelet count	363 ×10⁹/L	150–450 ×10⁹/L
Prothrombin time (PT)	15 s	12–16 s
International normalized ratio (INR)	1.1	0.9–1.3
Activated partial thromboplastin time (aPTT)	34 s	25–35 s
Serum sodium	136 mmol/L	135–145 mmol/L
Serum potassium	3.9 mmol/L	3.5–5.0 mmol/L
Serum chloride	102 mmol/L	98–107 mmol/L
Serum bicarbonate	20.4 mmol/L	20–28 mmol/L
Serum ionized calcium	1.20 mmol/L	1.15-1.29 mmol/L
Serum urea	22 mg/dL	10–40 mg/dL
Serum creatinine	0.52 mg/dL	0.4–0.9 mg/dL
Alkaline phosphatase (ALP)	110 U/L	70–390 U/L
Alanine aminotransferase (ALT)	8 U/L	7–35 U/L
Albumin	4.5 g/dL	3.5–5.0 g/dL
C-reactive protein (CRP)	4.7 mg/L	<5 mg/L
Hemoglobin electrophoresis	No abnormal variants detected	-
Thrombophilia screen	Negative	-

Cardiac evaluation with Holter monitoring, transthoracic echocardiography, and transesophageal echocardiography was unremarkable. Hemoglobin electrophoresis was normal. Vascular imaging was normal, ruling out vasculitis and dissection. Hypercoagulable states, connective tissue disorders, and drug and toxin ingestion were ruled out, and no clear etiology for her stroke was identified.

She remained hospitalized for several days for monitoring and initiation of physiotherapy, during which her symptoms gradually improved. In view of a cryptogenic stroke, whole exome sequencing (WES) was obtained. Although no definitive embolic source was identified, short-term anticoagulation was considered appropriate after multidisciplinary discussion. She was transitioned to rivaroxaban 15 mg once daily and discharged with ongoing rehabilitation.

At outpatient follow-up, her left-sided weakness had improved, with only mild upper-limb ataxia remaining. She was able to mobilize independently and resume daily activities. Proband WES revealed a heterozygous pathogenic (MB5) mutation (as classified by several clinical laboratories testing patients with ARVD), in PKP2 NM_0010054242.3, variant c.1237C>Tp.(Arg413*), with autosomal dominant inheritance. The parent of origin is unknown, as genetic testing was not done for the parents. This genetic mutation is associated with ARVD. She was referred to cardiology, where ECG, repeat echocardiography, and cardiac MRI were all normal. She did not meet clinical diagnostic criteria for ARVD.

The family was counseled regarding the genetic finding, and screening of first-degree relatives was recommended. A repeat brain MRI performed three months after presentation showed evolution to a chronic left superior cerebellar infarct with no new acute lesions. The patient was switched from rivaroxaban to aspirin and advised to continue it. She was advised to maintain annual follow-up visits with cardiology.

Regarding anti-thrombotic therapy, the patient was initially treated with aspirin at presentation. Following identification of a posterior circulation infarct with a non-occlusive basilar tip thrombus and concern for a potential cardioembolic mechanism, anticoagulation with rivaroxaban was initiated after multidisciplinary discussion, despite the absence of a definitive embolic source. Subsequent clinical stability, lack of recurrent events, normal extended cardiac evaluation, and follow-up imaging demonstrating chronic infarction without new lesions supported de-escalation back to antiplatelet therapy, and aspirin was resumed for long-term secondary prevention.

## Discussion

Ischemic stroke due to ARVD has rarely been reported in the literature. A case report by Parikh et al. describes an acute ischemic stroke as the presenting manifestation of ARVD in a young patient who was later found to have echocardiographic and cardiac MRI findings supporting the diagnosis. The authors suggest ARVD should be considered as a potential underdiagnosed cause of stroke in young individuals, emphasizing the importance of early diagnosis for management and prevention of sudden cardiac death [5].

Of note, ischemic strokes in children with cardiac disease frequently affect the anterior circulation, are often bilateral, and may demonstrate hemorrhagic transformation [6].

Another case report describes an embolic stroke in a young male caused by a loss-of-function SCN5A gene variant, which can lead to atrial myopathy, right ventricular abnormalities consistent with arrhythmogenic right ventricular cardiomyopathy, and can rarely lead to embolic stroke [7].

The only other reported case of stroke in association with ARVD was one in a case series, describing a 26-year-old male who had a history of unexplained ischemic stroke and was later found to have ARVD after workup for episodes of palpitations and anxiety [8].

Importantly, all previously reported cases had abnormal cardiac evaluations and were clinically diagnosed with ARVD. In contrast, our patient carries a pathogenic genetic variant associated with ARVD but has no cardiac manifestations to date. Of note, ARVD symptoms are most commonly recognized between the ages of 10 and 50 years, with an average age at diagnosis of about 30 years [9]. Therefore, our patient could still have been in the concealed stage of the disease and therefore had a normal cardiology workup.

Whether a pathogenic PKP2 mutation, in the absence of cardiac manifestations, can independently predispose an individual to stroke remains unclear. Potential mechanisms include occult or intermittent atrial or ventricular arrhythmias leading to transient cardioembolism, thrombus formation in the setting of early or concealed ventricular dysfunction, or broader arrhythmogenic cardiomyopathy phenotypes in which atrial involvement has been described. Additionally, while desmosomal proteins are primarily studied in myocardial tissue, their broader role in cellular adhesion raises speculative questions about extracardiac effects; however, such mechanisms remain unproven.

Posterior circulation stroke in children is frequently cryptogenic and may result from alternative mechanisms, including transient arteriopathy, unrecognized vertebrobasilar dissection (unlikely in our patient, as imaging findings were not suggestive), migraine-related phenomena, or prothrombotic states that evade detection. These considerations reinforce the possibility that the PKP2 variant identified in our patient may be incidental. The genetic finding is therefore presented as a potential modifier of risk and a driver of long-term cardiac surveillance rather than a definitive etiologic explanation for the stroke.

Given the absence of an identifiable etiology, the presence of a potentially arrhythmogenic genetic mutation, and the risk of recurrent events, a therapeutic decision was made to maintain the patient on aspirin and to arrange annual cardiology follow-up. Family screening was also recommended due to the autosomal dominant inheritance pattern of PKP2-related disease.

## Conclusions

This case highlights the diagnostic challenges inherent in evaluating pediatric ischemic stroke, particularly when standard investigations fail to reveal an underlying cause. The identification of a pathogenic PKP2 variant in our patient, despite normal cardiac evaluation and the absence of clinical features of ARVD, raises important questions about the potential cerebrovascular relevance of desmosomal gene mutations. Although the relationship between PKP2 variants and stroke remains uncertain, the presence of a pathogenic mutation warrants careful long-term cardiac surveillance and consideration of antithrombotic therapy.

Our patient was maintained on aspirin for secondary stroke prevention with the intent of long-term therapy, given the cryptogenic nature of the event and absence of an alternative etiology. Escalation to anticoagulation would be reconsidered if future cardiac surveillance were to identify clinically significant arrhythmias, intracardiac thrombus, or other features suggestive of increased cardioembolic risk, in line with standard pediatric stroke and cardiology practice. The patient and the family were advised to continue routine daily activities, with avoidance of competitive or high-intensity sports pending longitudinal cardiology follow-up. This case underscores the value of comprehensive assessment, including genetic testing, in children presenting with unexplained stroke and highlights the need for further research into the broader phenotypic spectrum of PKP2-associated disease. Larger, longitudinal studies are needed to determine whether PKP2 variants confer any independent cerebrovascular risk and to guide evidence-based management.
